# The life cycle of voltage-gated Ca^2+^ channels in neurons: an update on the trafficking of neuronal calcium channels

**DOI:** 10.1042/NS20200095

**Published:** 2021-02-23

**Authors:** Laurent Ferron, Saloni Koshti, Gerald W. Zamponi

**Affiliations:** Department of Physiology and Pharmacology, Alberta Children’s Hospital Research Institute, Hotchkiss Brain Institute, Cumming School of Medicine, University of Calgary, Calgary, AB, Canada

**Keywords:** calcium channel, internalization, trafficking

## Abstract

Neuronal voltage-gated Ca^2+^ (Ca_V_) channels play a critical role in cellular excitability, synaptic transmission, excitation–transcription coupling and activation of intracellular signaling pathways. Ca_V_ channels are multiprotein complexes and their functional expression in the plasma membrane involves finely tuned mechanisms, including forward trafficking from the endoplasmic reticulum (ER) to the plasma membrane, endocytosis and recycling. Whether genetic or acquired, alterations and defects in the trafficking of neuronal Ca_V_ channels can have severe physiological consequences. In this review, we address the current evidence concerning the regulatory mechanisms which underlie precise control of neuronal Ca_V_ channel trafficking and we discuss their potential as therapeutic targets.

## Introduction

**Figure 1:**
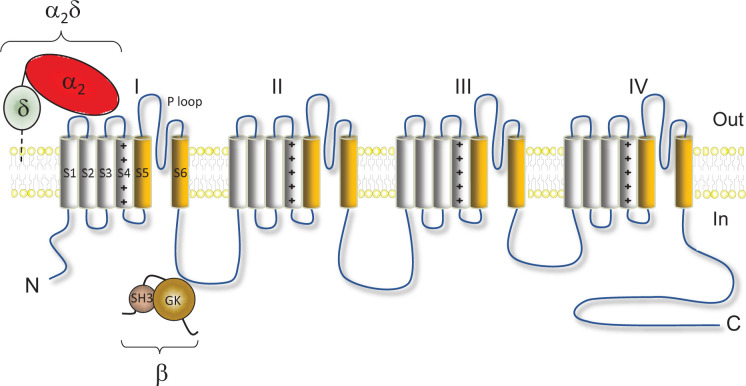
Schematic representation of the structure of Ca_V_ channels The Ca_V_α_1_ subunit is formed by four repeat domains (I–IV) each containing six transmembrane segments: S1–S4 constitute the voltage sensor domain (S4 segments contain positively charged residues) and S5–S6 constitute the pore domain (the P loops contain acidic residues that contribute to the selectivity filter of the channel). Ca_V_α_1_ subunits can be associated with auxiliary subunits: an extracellular α_2_δ subunit attached to the plasma membrane by a glycosyl phosphatidylinositol (GPI) anchor and an intracellular β subunit which contain a src homology 3 (SH3) domain and a GK domain.

Calcium (Ca^2+^) channels mediate numerous important physiological processes, and are abundant in many types of cells [1,2]. In neurons, voltage-gated Ca^2+^ (Ca_V_) channels are expressed in most plasma membrane compartments and they are involved in regulating cell excitability, gene transcription and synaptic transmission. Ca_V_ channels are activated by membrane depolarization and they can be classified into two major categories: high-voltage-activated channels (HVAs), consisting of L-type (Ca_V_1.1, 1.2, 1.3 and 1.4), P/Q-type (Ca_V_2.1), N-type (Ca_V_2.2), and R-type (Ca_V_2.3) channels, and low-voltage-activated channels (LVAs), which encompass the T-type channels (Ca_V_3.1, Ca_V_3.2, Ca_V_3.3) [3,4]. All HVA channels contain multiple subunits which assemble to form a functional channel complex (Figure 1). These subunits include the pore forming Ca_V_α_1_ subunit and auxiliary α_2_δ and β subunits, and in some cases a γ subunit. Conversely, LVA channels only require a Ca_V_α_1_ subunit to be functional.

Pore forming Ca_V_α_1_ subunits exhibit four repeat domains each containing six transmembrane segments (Figure 1). Crystallography and cryo-EM experiments have provided exquisite details of the atomic structure of Ca_V_ channels and their auxilliary subunits [5–7]. Segments S1–S4 constitute the voltage-sensing domain and segments S5–S6 form the pore and the selectivity filter. The amino (N) and carboxy (C) termini and the cytoplasmic loops that connect the four transmembrane domains are important domains involved in the modulation of the activity of the channels, as well as forming critical protein interaction platforms that regulate the trafficking of Ca_V_ channels to the plasma membrane.

Auxilliary β subunits are crucial for the regulation of HVA channel activity through modulation of their biophysical properties [8–11] and the control of their membrane trafficking [8,11–13]. There are four different types of β subunits (encoded by four genes) and they are largely cytoplasmic. However, palmitoylation of the β_2a_ subunit takes place post-translationally at its N-terminus and results in the targeting of the subunit to the plasma membrane [14]. All β subunits consist of five distinct structural regions: the N-terminus, the src homology 3 (SH3) domain, the HOOK domain, the GK domain, and the C-terminus [8,15]. The GK and SH3 domains are highly conserved across the different β subunits, and are connected by a variable HOOK domain. The effects of β subunits on HVA channels are mediated by the GK domain, through a region termed the α Interaction Domain (AID) Binding Pocket (ABP) [16–18]. The ABP binds to a region called the AID domain in the I–II loop of the Ca_V_α_1_ subunit, which contains several key residues that modulate β subunit binding. However, it has also been reported that β subunits most likely interact with other regions of Ca_V_α_1_ subunits [19]. β subunits can bind Ca_V_α_1_ subunits in the endoplasmic reticulum (ER) prior to processing in the Golgi, and the resulting Ca_V_α_1_-β subunit complex is often found to be localized at the plasma membrane [9,20].

Auxilliary α_2_δ subunits are also critical for the trafficking of HVA channels [3,11,21]. There are four different genes ecoding α_2_δ subunits, namely α_2_δ-1 to -4 [22]. α_2_δ subunits are extracellular proteins, translated into one precursor that is post-translationaly proteolytically cleaved into α_2_ and δ peptides which remain attached by disulfide bonds [23,24]. The δ part of α_2_δ was initially predicted to be a transmembrane protein but it was later demonstrated that δ remains attached to the extracellular leaflet of the plasma membrane by a glycosylphosphatidylinositol (GPI) anchor [7,25–27]. α_2_δ subunits contain different functional domains: a von Willebrand factor A (VWA) domain with a metal ion-dependent adhesion site (MIDAS) and multiple Cache domains [7,28]. Several regions of α_2_δ have been predicted to interact with Ca_V_ channels based on a cryo-EM study [7] and some of these putative interactions have recently been validated by functional studies [29,30].

**Figure 2:**
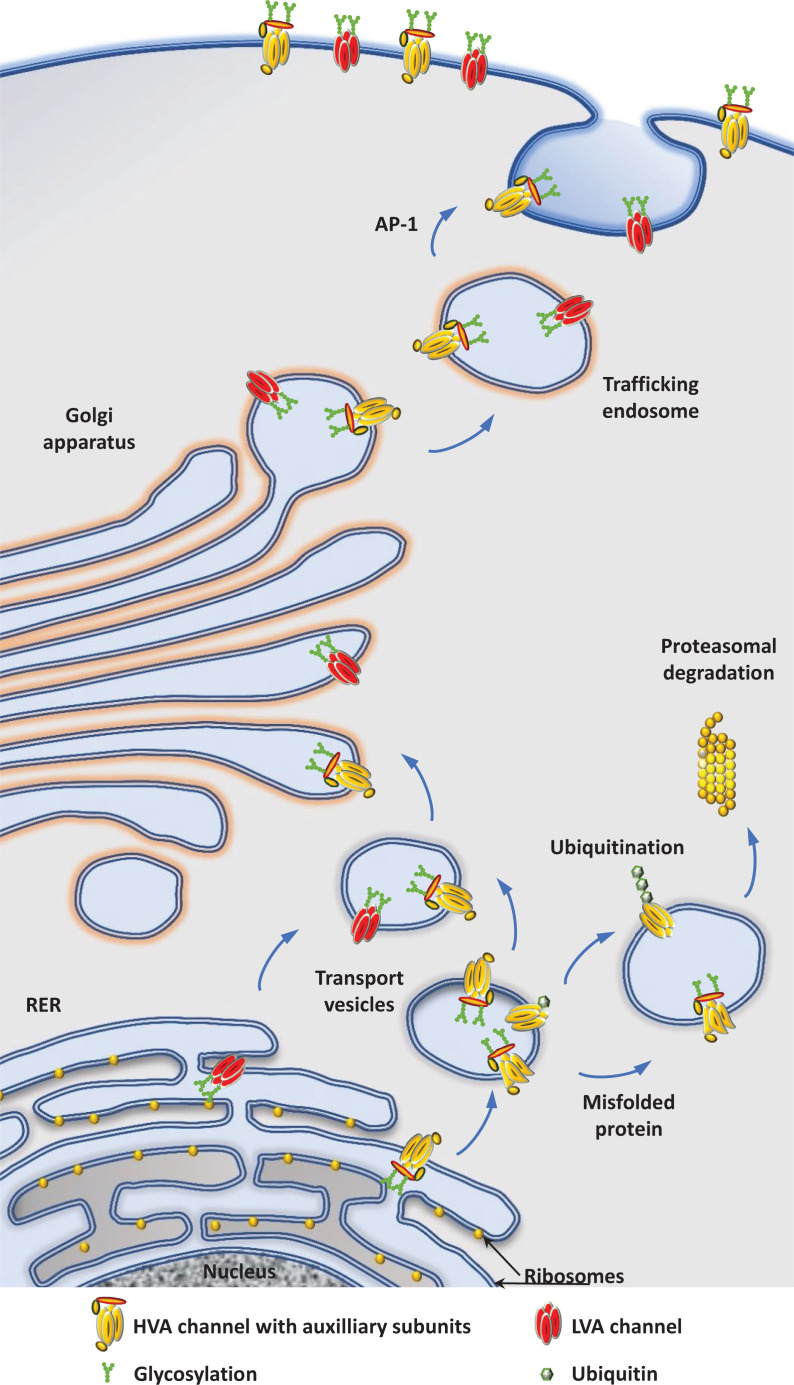
Diagram of forward trafficking mechanisms of Ca_V_ channels from the ER to the plasma membrane Newly synthesized peptides are translocated to the rough ER (RER) where they associate with auxiliary subunits and are subjected to post-translational modifications including glycosylation. Ca_V_ channels are then trafficked to the plasma membrane via the Golgi apparatus and trafficking endosomes. Along the way, misfolded proteins are identified by quality-control mechanisms and targeted for degradation. The association of Ca_V_α_1_ with β subunits prevents the ubiquitination of the Ca_V_α_1_ subunit which protect channels from degradation by the proteasome. The adaptor protein AP1 interacts with Ca_V_α_1_ and contribute to the incorporation of channels to the plasma membrane via clathrin-coated vesicles.

**Figure 3:**
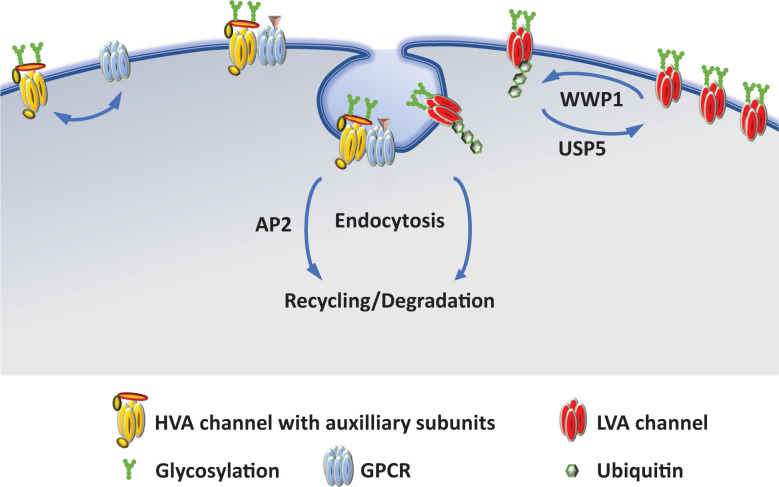
Schematic depiction of the internalization of Ca_V_ channels The stability of Ca_V_ channels at the plasma membrane is determined by the activity of the channel and by the interaction with regulatory proteins. G protein-coupled receptors (GPCRs), like the D2R dopamine receptor, have been shown to directly interact with Ca_V_2.2 channels and to induce the internalization of the complex when the receptor is activated by its agonist. The adaptor protein 2 (AP2) has been implicated in this internalization process. For Ca_V_3.2, the balance between ubiquitination/de-ubiquitination is key to the stability of the channels in the plasma membrane. USP5, a de-ubiquitinase, removes ubiquitin from Ca_V_3.2 increasing the lifetime of the channels at the plasma membrane whereas WWP1, a ubiquitin ligase, transfers ubiquitin to Ca_V_3.2 and promotes the endocytosis of the ubiquitinated channels. Endocytosed Ca_V_ channels are then either recycled or degraded.

**Figure 4:**
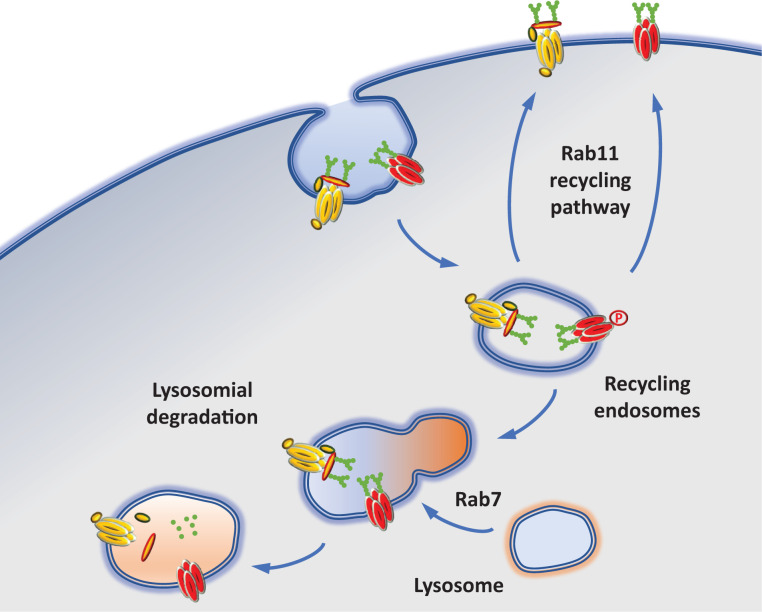
Schematic of the recycling and degradation of Ca_V_ channels Endocytosed Ca_V_ channels are either recycled or degraded. Rab11, a small GTPase that controls key events of vesicular transport, is suspected to be a major player in the recycling of Ca_V_ to the plasma membrane by interacting either with the Ca_V_α_1_ subunit or with the α_2_δ auxiliary subunit. Following their endocytosis Ca_V_ channels have been shown to be co-localized with Rab7, a marker for late endosomes and lyzosomes.

Ca_V_ channel subunits are synthesized by ER-bound ribosomes and inserted into the ER membrane while being synthesized. From the ER membrane, proteins are then trafficked to the plasma membrane via the Golgi network and trafficking endosomes (Figure 2). During their journey to the plasma membrane, Ca_V_α_1_ subunits undergo maturation steps and quality control checks, including association with auxiliary subunits β and α_2_δ, that affect their ability to reach the plasma membrane and fulfill their physiological roles. Once at the plasma membrane, the fate of Ca_V_ channels is determined by the dynamic interactions with anchoring proteins, binding partners and the activity of the neurons. Ca_V_ channels can then be internalized and either recycled or degraded (Figures 3 and 4). In this review, we will highlight our current knowledge about the trafficking of neuronal Ca_V_ channels with a focus on the mechanisms that regulate these processes.

## Forward trafficking of Ca_V_: from ER to plasma membrane

### Glycosylation of the Ca_V_α_1_ subunit

Glycosylation in the ER and the Golgi system contributes to the quality control of protein folding [31–33]. N-linked glycosylation corresponds to the transfer of oligosaccharide chains (glycans) on to asparagine residues of newly synthesized proteins in the ER. The N-glycans interact with lectin chaperones such as calnexin and calreticulin to ensure the selective export of properly folded proteins. Although a critical role of N-glycosylation on the trafficking and function of membrane proteins such as ion channels has first been demonstrated over a decade ago [34–41], it was only recently that its impact on Ca_V_ channels (mainly Ca_V_3.2 T-type channels) has attracted more attention [42,43]. Four putative N-glycosylation sites have been identified in extracellular loops of Ca_V_3.2 channels: N192 in loop 2 of domain I; N271 in loop 3 of domain I; N1466 in loop 3 of domain III; N1710 in loop 2 of domain IV [44,45]. A combination of pharmacological tools and site-directed mutagenesis was used to characterize the role of these glycosylation sites: residues N271 and N1710 are essential for passing quality control as their mutations induce an almost complete loss of protein expression. Although discrepencies have been reported regarding the magnitude of the effect of mutating N1466 and N192 on Ca_V_3.2 functional expression, it appears that these glycosylation sites are critical for the trafficking of the channels to the plasma membrane and that they also affect the biophysical properties of the channels [44–46]. A recent study has investigated the impact of the double mutation N192Q and N1466Q on the trafficking of Ca_V_3.2 by scrutinizing lateral mobility (investigated by fluorescence recovery after photobleaching) and internalization (investigated by antibody internalization assay) [47]. These data revealed that whereas lateral mobility is not affected, the internalization rate of Ca_V_3.2 channels is increased when the glycosylation sites are mutated indicating that the stability of channels at the plasma membrane is reduced [47]. However, further investigations focusing on the net forward trafficking of Ca_V_3.2 will be needed to ascertain a role of N-glycosylation of Ca_V_3.2 on its recycling or forward trafficking. As Ca_V_3.2 channel up-regulation is a common feature in the development and maintenance of multiple pain processes [48], and alterations of Ca_V_3.2 channels glycosylation have been associated with the development of pain related to diabetes [43,45,49], understanding Ca_V_3.2 trafficking and the impact of its glycosylation are of significant therapeutic relevance [50].

### Auxiliary α_2_δ subunits

The α_2_δ subunits associate with Ca_V_α_1_ subunits in the ER and promote their trafficking to the plasma membrane [3,4]. However, the exact mechanism by which α_2_δ increases the density of Ca_V_ channels at the plasma membrane is still under investigation. Evidence obtained from a neuronal cell line (N2a cells) transiently expressing Ca_V_2.2 indicated that α_2_δ-1 does not affect the endocytosis of the channel [51]. Instead, α_2_δ subunits are suspected to control the trafficking of Ca_V_ channels either by promoting their transfer from the ER to the plasma membrane or by increasing their recycling.

The α_2_δ subunits are also synthesized by ER-bound ribosomes and translocated in the ER lumen. α_2_δ subunits are highly glycosylated [26,52] and this process is critical for the trafficking of Ca_V_ channels. It then appears obvious that, as the glycosylation state of α_2_δ subunits affects their trafficking to the plasma membrane, it can consequently affect the trafficking of the pore forming unit [53,54]. Tetreault and colleagues performed an extensive site-directed mutagenesis study of the 16 putative N-glycosylation sites of α_2_δ-1 and showed that, in addition to playing a role in stability/quality control and trafficking of α_2_δ-1, specific glycosylation sites of α_2_δ-1 are involved in the modulation of Ca_V_1.2 biophysical properties [53].

The α_2_δ-1 subunit was shown to interact with the low-density lipoprotein receptor-related protein-1 (LRP1) [55]. When LRP1 is expressed with its chaperone protein, the receptor-associated protein (RAP), it promotes α_2_δ-1 glycosylation maturation, trafficking, and cell surface expression. This LRP1/RAP complex also promotes the functional expression of Ca_V_2.2 (cell surface expression and current density).

Besides glycosylation, α_2_δ is subject to additional post-translational modifications such as the formation of disulfide bonds and the proteolytic cleavage [56]. Disulfide bonds allow α_2_ to stay linked to δ and thus to the membrane after the proteolytic cleavage. The proteolytic cleavage of α_2_δ does not appear to affect the trafficking of Ca_V_2 channels but plays a role in the fully functional channel complex [57,58].

### Auxiliary β subunits

It is well established that β subunits have a direct role in trafficking HVA (Ca_V_1.X and Ca_V_2.X) channels to the plasma membrane. However, the mechanism of how this occurs is yet to be fully elucidated. Initially, it was reported in *Xenopus laevis* oocytes that β subunits co-expressed with Ca_V_2.1 channels resulted in an increase in Ca^2+^ current amplitude [9]. It was hypothesized that β subunit binding to Ca_V_2.1 α_1_ resulted in the masking of an ER retention motif present on the I–II loop [12,59]. However, studies performed on Ca_V_1.2 and Ca_V_2.2 did not provide evidence that such an ER retention signal exists in their I–II loop. Indeed, CD4 proteins fused to the I–II linker of Ca_V_1.2 or Ca_V_2.2 are efficiently trafficked to the plasma membrane in the absence of β subunits [60]. Furthermore, chimeric channels formed by swapping the I–II linkers from Ca_V_1.2 or Ca_V_2.2 to Ca_V_3.1 α_1_ subunits, which do not require β subunits for their plasma membrane targeting, generated larger currents than wild type Ca_V_3.1 [61,62]. Altogether, these studies support the existence of an ER export signal in the I–II loop of Ca_V_1.2 and Ca_V_2.2. Finally, extensive analysis of Ca_V_1.2 intracellular domains identified ER retention signals in all the other intracellular linkers and in the N- and C-termini [13,60,61]. Current thinking is that when a β subunit binds the the AID of a Ca_V_α_1_ subunit in the ER, conformational changes mask the retention signals and expose the export signal. This then allows the channel complex to be trafficked to the plasma membrane [8,15]. However, questions remain about the function of the I–II linker (ER retention or export signal) between Ca_V_2.1 and the other HVA channels [59–61]. For example, does this difference point to a Ca_V_2.1 channel specificity? Further investigation will be needed to confirm this speculation.

β subunits increase the trafficking of Ca_V_ channels by playing the role of a trafficking switch but it was also shown that they can prevent the degradation of Ca_V_ channels by the proteasome [60,63]. In heterologous expression systems, β subunits reduce Ca_V_1.2 degradation by binding to the AID domain and inhibiting its ubiquitination by the E3 ubiquitin ligase RFP2 [60]. In the absence of β subunits, ubiquitinated Ca_V_1.2 channels interact with the ER-Associated Degradation (ERAD) complex derlin-1/p97 proteins to be targeted to the proteasome for degradation. The role of RFP2, and hence ubiquitination, in controlling Ca_V_1.2 trafficking to the plasma membrane was confirmed in hippocampal neurons [60]. Similarly, for Ca_V_2.2 channels it was shown in rat superior cervical ganglia (SCG) neurons that a mutation in the AID domain that prevents the binding of β subunits [64] induced an increase in the channel degradation compared with wildtype Ca_V_2.2. This effect was blocked by proteasomal inhibitors [63]. It was later shown that the interaction with β subunits prevents the poly-ubiquitination of the Ca_V_2.2 I–II loop and its proteasomal degradation, thus increasing the forward trafficking of the channel [65].

Nedd4-1, a ubiquitin ligase, was reported to decrease the plasma membrane density of Ca_V_1.2 in a β-dependent manner through lysosomal degradation [66]. However, the mechanism of action of Nedd4-1, which does not involve a direct ubiquitination of the channel complex, remains to be elucidated.

Furthermore, the phosphorylation state of β subunits can affect Ca_V_ channel trafficking to the plasma membrane. In COS7 cells and rat dorsal root ganglion (DRG) neurons, Akt, a kinase in the PI3Kγ pathway, was reported to phosphorylate β subunits through a PIP3-dependent mechanism [67]. Akt specifically phosphorylates a serine residue in the C-terminus of β2a leading to an increase in trafficking of the channels (Ca_V_1.2 and Ca_V_2.2) to the plasma membrane and an increase in calcium current density [67]. The effect of Akt on Ca_V_1.2 was later shown to occur also in cardiomyocytes [68]. Altogether, these studies suggest that the phosphorylation of β2a promotes its chaperone role on Ca_V_ channels.

As we will discuss in the Endocytosis section of this review, G protein-coupled receptors (GPCRs) are potent modulators of Ca_V_ channel trafficking to the plasma membrane through direct interaction with the Ca_V_α_1_ pore-forming subunit. However, the Growth Hormone Secretagogue Receptor type 1a (GHSR), a GPCR that constitutively controls Ca_V_ current density via G_i/0_ activation, was recently shown to exert its effect by promoting the retention of Ca_V_ channels in the ER [69,70]. Intriguingly, this effect of GHSR on Ca_V_ channels depends on the presence of β subunits but does not rely on the interaction of β with the AID of Ca_V_ channels. Further studies will be needed to identify the molecular mechanism at play in this signaling pathway.

### Adaptor protein 1

From the surface of the trans-Golgi network, clathrin-coated vesicles are formed by the recruitment of clathrin via heterotetrameric Adaptor Protein 1 (AP1) complexes [71]. Clathrin-coated vesicles are responsible for the transport of cargo molecules to the plasma membrane. Membrane-bound AP1 complexes interact with sorting signals (YxxΦ and [DE]xxxL[LI], where x is any amino acid and Φ is a bulky hydrophobic residue) contained within the cytosolic tails of transmembrane proteins. Such sorting signals have been identified in the proximal C-terminus of Ca_V_2.2 [72]. The mutation of these consensus motifs in Ca_V_2.2, the knockdown of one component of the AP1 complex (AP1 γ) using shRNA, and the expression of a dominant negative form of one component of AP1 complex (AP1 σ) all reduced the cell surface expression of Ca_V_2.2 in N2a cells and in DRG neurons. These findings demonstrate the functional involvement of the AP1 complex in the trafficking of Ca_V_2.2 channels to the plasma membrane [72]. AP1 binding motifs are located in exon 37 of Ca_V_2.2. Exon 37 is subject to alternative splicing and can generate 2 mutually exclusive variants (37a and 37b) [73,74]: exon 37a contains two AP1 consensus sites whereas exon 37b contains only one noncanonical AP1 site [72]. It is worth noting that cell surface expression of Ca_V_2.2 channels containing exon 37a is higher than Ca_V_2.2 channels containing exon 37b which reinforces the importance of this region for the trafficking of Ca_V_2.2 to the plasma membrane. Moreover, exon 37a is selectively expressed in peripheral nociceptive neurons and its expression is critical for pain signaling [73,75]. AP1 consensus binding motifs can also be found in the proximal C-terminus of Ca_V_1.3, Ca_V_1.4 and Ca_V_2.1 (exon37a) which suggests that forward trafficking of these channels may also be AP1 dependent. Altogether, these data highlight the possibility that targeting AP1/Ca_V_2.2 interactions may serve as a therapeutic approach towards pain modulation.

### Fragile X mental retardation protein

The Fragile X mental retardation protein (FMRP) was shown to control the functional expression of ion channels [76–78]. FMRP affects Ca_V_2.2 channels in neurons by directly interacting with intracellular domains of Ca_V_2.2, including its C-terminus [79]. In a recent study (using Ca_V_2.2 channels with a tandem α-bungarotoxin binding site (BBS) tag in an extracellular loop expressed in N2a cells), FMRP was shown to reduce the trafficking of the channels between the Golgi network and the plasma membrane [80]. Although the exact binding domain of FMRP on the C-terminus of Ca_V_2.2 still has to be identified, it is possible that FMRP interferes with the binding of the AP1 complex to the Ca_V_2.2 C-terminus, thereby affecting its forward trafficking as a consequence.

### Stac proteins

The Stac3 (SH3- and cysteine-rich domains) protein is essential for EC coupling in skeletal muscle [81,82]. The functional interaction between Stac3 and Ca_V_1.1 induces an increase in channel density in the plasma membrane and alters the kinetics of the Ca_V_1.1-generated current in tsA-201 cells [83]. Stac proteins were also shown to alter the Ca^2+^-dependent inactivation of neuronal L-type channels Ca_V_1.2 and Ca_V_1.3, however Stac proteins have no effect on the trafficking of these channels [84,85]. Finally, whereas no effect were reported on non L-type channels (Ca_V_2.1), Stac1 was shown to increase the expression of Ca_V_3.2 [86]. Further studies will be needed to determine whether Stac proteins increase the forward trafficking or the stability of these channels at the plasma membrane.

### Truncated channels and mutation of the Ca^2+^-binding site in the pore

Genes encoding Ca_V_α_1_ subunits are transcribed into pre-messenger RNA that is subject to cell specific and developmentally regulated alternative splicing [73,87–92]. Splicing of Ca_V_α_1_ subunits has the ability to generate a multitude of full-length fully functional channels. However, alternative splicing can also give rise to truncated proteins with altered or no channel activity. Functional studies performed on Ca_V_1.1, Ca_V_1.2 and Ca_V_2.1 have shown that truncated channels have physiological relevance by controlling the expression of full-length Ca_V_ channels [93–96]. Moreover, mutations that result in truncations of Ca_V_α_1_ subunits are suspected to cause pathological states. For example, in episodic ataxia type-2 (EA-2), an autosomal dominant disorder, mutations in the gene *CACNA1A* that encodes Ca_V_2.1 predict truncated forms of this channel [97,98]. The expression of a truncated channel, either physiological or pathological, was shown to have a dominant-negative effect on co-expressed full-length channels [95,98]. Indeed, it has been shown that truncated Ca_V_2.2 and Ca_V_2.1 subunits interact with the full-length channels in the ER. The complex is then either recognized as misfolded proteins which activates a component of the unfolded protein response (UPR) inducing translational arrest [99,100] or targeting for degradation by the proteasome [101]. The N-terminus of the channel is key for the interaction between truncated and wildtype channels and disrupting this interaction has been considered as a potential therapeutic intervention [102,103].

A recent study has investigated the role of the selectivity filter of Ca_V_2.1 and Ca_V_2.2 in the trafficking of the channels to the plasma membrane [104]. This study shows that Ca^2+^-binding sites in the selectivity filter have to be preserved for the channel to be optimally trafficked to the plasma membrane and the authors hypothesized that Ca^2+^ binding to the pore is required for the proper folding of the channel in the ER and therefore for its trafficking.

### Calmodulin

The role of calmodulin (CaM) in the regulation of Ca^2+^-dependent inactivation of Ca_V_ has been extensively studied [105]. However, CaM involvment in the trafficking of Ca_V_ remains unclear. CaM is able to bind several motifs in the C-terminus of Ca_V_1.X and Ca_V_2.X channels [105] and the deletion of these CaM binding motifs in Ca_V_1.2 was shown to abolish the cell surface expression of the channels [13] and to alter Ca_V_1.2 current amplitude [106–108] suggesting that CaM can modulate the trafficking of Ca_V_1.2 channels to the plasma membrane. However, a more recent study challenged this conclusion [109]. Bourdin and colleagues used tsA-201 cells to express an extracellularly tagged Ca_V_1.2 and examined the effect of CaM and a dominant negative CaM on its cell surface expression [109]. They quantified Ca_V_1.2 plasma membrane expression by using fluorescence-activated cell sorting analysis. They did not observe an effect of CaM on Ca_V_1.2 cell surface expression, thus concluding that CaM is not essential for the trafficking of Ca_V_1.2 channels [109]. Overall, it can be argued that deletions and/or mutations in the C-terminus of Ca_V_1.2 channel can affect its trafficking either by disrupting trafficking signals, for example an ER export signal [60], or by affecting the folding of the protein [104], and caution should be exerciced when interpreting the results of such experiments.

Fully mature channels reach the plasma membrane as a protein complex formed by a main Ca_V_α_1_ subunit and auxiliary subunits, glycosylated and associated with binding partners. These complexes are now able to play their physiological role in letting Ca^2+^ flow inside the cell, modulating the excitability of the neurons and activating signaling pathways. Their lifetime at the plasma membrane is then dictated by the cell’s activity, the stability of the interactions with their existing partners and the interactions with new ones.

## Endocytosis and recycling of Ca_V_

### GPCRs

GPCRs play critical roles in modulating the activity of Ca_V_ channels [4]. GPCRs have been described as part of signaling complexes together with Ca_V_s, including Ca_V_1.2 and β-2 adrenergic receptors [110,111], Ca_V_2.1 and mGluR1 [112], Ca_V_2.2 and opioid receptors, dopamine receptors (D1R and D2R), GABA_B_ receptors, and MT1 melatonin receptors [113–120]. GPCRs activated by their specific agonist bind to a heterotrimeric G protein. This is followed by the exchange of GDP for GTP and dissociation of the G protein into GαGTP and Gβγ. G protein-mediated regulation of Ca_V_ channels affects their biophysical properties [4,121–124]. For example, Gα(s)-GTP activated by β-2 adrenergic receptors in neurons triggers a cAMP/PKA cascade which culminates in an increase in L-type currents [110,125]. The Gβγ dimer can also trigger specific downstream events including the modulation of Ca_V_ channel activity. Indeed, Gβγ has been shown to directly interact with intracellular domains of the Ca_V_2.X family [126,127] and Ca_V_3.2 channels [128,129]. For Ca_V_2.X channels, Gβγ interacts with the I–II loop and the N-terminus domain and it induces voltage-dependent inhibition [126,127]. For Ca_V_3.2 channels, Gβγ interacts with the II–III loop and reduces the open probability of the channel [128,129].

While G protein-mediated effects of GPCRs modulate the biophysical properties of Ca_V_ channels, receptors themselves, including ORL1, D1R and D2R, have been shown to control the cell surface expression of the channels, and this can occur through both ligand-independent and ligand-dependent effects. For the ligand-independent effect, the co-expression of several types of GPCRs (ORL1, D1R and D2R) has been reported to increase the number of Ca_V_2.2 channels at the plasma membrane [72,114,116,117]. For D1R and ORL1, the interaction with Ca_V_2.2 occurs through direct binding of intracellular regions of the receptors with the proximal C-terminus of the channels [113,116]. Although they still have to be experimentally demonstrated, several mechanisms have been proposed to explain the increase in channel plasma membrane expression: the receptors could mask an ER retention signal contained within the C-terminus of Ca_V_2.2 [60] and/or the receptor itself could confer an additional trafficking motif to the channel complex. Moreover, a D2R-dependent increase in Ca_V_2.2e37b cell surface expression in N2a cells has been linked to a reduction in the rate of endocytosis [72]. The molecular mechanism involved in this latter effect has yet to be identified, however, as it only occurs for e37b and not e37a, this suggests the presence of a specific interaction motif with D2R within protein sequence encoded by exon 37b. For the ligand-dependent effect, the activation of the receptor induces the internalization of the receptor/channel complex. This effect was shown for ORL1, D1 and D2 receptors [72,114,116,117]. Interestingly, due to the ability of ORL1 to heterodimerize with opioid receptors [130], activated opioid receptors are also able to co-internalize with Ca_V_2.2 channels when they are co-expressed with ORL1 [115]. The mechanism of internalization of the complex has not yet been fully elucidated. Nonetheless, for D2R and Ca_V_2.2, the internalization of the activated complex relies on both the AP2μ2 protein and an AP2 binding motif in the C-terminus of Ca_V_2.2 which suggests a clathrin-mediated endocytosis via β-arrestin [72].

### RGK proteins

RGK GTPases are a family of small GTPases consisting of Rem, Rem2, Rad and Gem/Kir [131–134] and they all have been shown to inhibit Ca_V_1.X and Ca_V_2.X channels [8,135]. RGK proteins can utilize multiple mechanisms to inhibit Ca_V_1.X and Ca_V_2.X channels: they can affect the channel’s cell surface expression, their open probability and they can immobilize the voltage sensor of the channel [135,136]. The respective contribution of each mechanism to the inhibitory effect of RGK on Ca_V_1.X and Ca_V_2.X is thought to be dependent on the combination RGK/channel types that a cell expresses [135]. Precisely how RGK proteins affect the trafficking of Ca_V_ channels is still not fully understood.

The first evidence of an inhibitory effect of RGK proteins on Ca_V_ channels was presented by Béguin and colleagues [137]. These authors identified Gem as a binding partner for β subunits (β_1_, β_2_ and β_3_) and then showed that the expression of Gem in *Xenopus* oocytes virtually abolished the currents generated by both Ca_V_1.2 and Ca_V_1.3 when co-expressed with β_1_, β_2_ or β_3_ subunits. Finally, they correlated the reduction in Ca^2+^ current with a reduction in Ca_V_1.2 cell surface expression. Indeed, they showed that the co-expression of Gem with Ca_V_1.2/β-3 in HEK 293 cells prevents Ca_V_1.2 channels from reaching the plasma membrane and this leads to the formation of intracellular channel aggregates. These results suggested that Gem competed with Ca_V_α_1_ subunits for binding of β subunits and consequently prevented Ca_V_α_1_ subunits from being trafficked to the plasma membrane. However, this hypothesis was challenged by a subsequent study by Yang and Colecraft [136] who investigated the inhibitory mechanism of Rem on Ca_V_1.2 channels co-expressed with β_2a_. The authors generated a Ca_V_1.2 channel tagged with an α-bungarotoxin binding site in an extracellular loop to monitor its cell surface expression in HEK 293 cells. They used a combination of biotinylated α-bungarotoxin and streptavidin coupled to quantum dots to show that the expression of Rem reduced Ca_V_1.2 cell surface expression by activating a dynamin-dependent mechanism. This suggested that Rem affects Ca_V_1.2 surface expression by increasing its internalization. They also showed that Rem-dependent internalization relied on the interaction with β_2a_ since the reduction in Ca_V_1.2 surface expression was not as noticeable in the absence of the β subunit. In subsequent studies, it was shown that RGK proteins can exert both β-binding-dependent and β-binding-independent inhibition of Ca_V_ channels [138–140]. Indeed, when a mutant β_2a_ subunit lacking the ability to bind Rem is co-expressed with Ca_V_1.2 and Rem in HEK 293 cells, Ca_V_1.2 current is still reduced [140]. Conversely, mutant Rem and Rad constructs that do not interact with β subunits are still able to inhibit Ca_V_1.2 currents [139]. Rem-dependent internalization of Ca_V_1.2 channels was shown to be due to a β-binding-dependent mechanism [140].

For years the mechanism by which β-adrenergic receptor activation increases whole cell L-type calcium currents has been subject to intense investigation and debate. It was recently uncovered in cardiomyocytes that Rad can be phosphorylated by protein kinase A (PKA). This causes a disruption of the interaction between Rad and β-subunits and relieves Rab-dependent inihibition of Ca_V_1.2 channels [141], thus giving rise to larger L-type currents. This β-binding-dependent inhibition of Rad affects the gating of Ca_V_1.2 channels rather than their trafficking to the plasma membrane [141,142]. PKA activation is triggered in cardiomyocytes by a β-adrenergic receptor pathway and plays a crucial role in the fight-or-flight response [143,144]. A similar PKA-dependent phosphorylation effect was demonstrated between Rad (and Rem) and Ca_V_1.3 and Ca_V_2.2 channels expressed in HEK 293T cells [141]. It would be of great interest to determine whether similar mechanisms occur in neurons.

The development of genetically encoded Ca_V_ channel inhibitors has been one of the foci of the Colecraft lab for many years [145]. Understanding the mechanisms by which RGK proteins inhibit Ca_V_ channels has allowed them to engineer RGK proteins that specifically inhibit subtypes of Ca_V_ channels in cardiomyocytes and in neurons, i.e. Ca_V_1.2 and Ca_V_2.2 [139]. In cardiomyocytes, it was shown that by targeting Rem expression to caveolae, only Ca_V_ channels localized to caveolae were inhibited, leaving Ca_V_1.2 channels responsible for excitation–contraction coupling in the T-tubule virtually unaffected [146]. Would a similar subcellular targeting strategy of RGK proteins be a means for inhibiting specific neuronal subtypes of Ca_V_? This could provide a powerful tool to tune synaptic transmission by targeting presynaptic Ca_V_2.2 channels without affecting somatic Ca_V_1.2 channels.

### Other interactors affecting Ca_V_ channel endocytosis

While the forward trafficking effects of β subunits have been under continuous inquiry, whether β subunits have a role in endocytosis of Ca_V_1.2 channels has yet to be thoroughly explored. A study by Hidalgo and colleagues first showed that the SH3 domain of the β subunit can increase the internalization of Ca_V_1.2 in *Xenopus* oocytes through a dynamin-dependent interaction [147]. They later found that homodimerization of the β-SH3 domain was necessary for Ca_V_1.2 endocytosis [148]. The endocytosis of Ca_V_1.2 occurs through the channel binding to a polyproline motif on the dynamin. Thus, there is evidence that the SH3 domain of β subunits has a role in modulating the endocytosis of Ca_V_, however further research is required to determine the net impact on Ca_V_1.2 surface expression when considering the forward trafficking effect of full-lenght β subunits.

In hippocampal neurons, α-actinin, which binds to F-actin, was shown to stabilize Ca_V_1.2 channels at the plasma membrane by preventing their endocytosis [149]. In resting conditions, α-actinin and apo-CaM (Ca^2+^-free CaM) both bind to site in the C-terminus domain of Ca_V_1.2 (IQ CaM binding domain) [144,149,150]. During prolonged activity, the influx of Ca^2+^ increases the affinity of CaM for the C-terminus Ca_V_1.2 and displaces the binding of α-actinin, thereby initiating the endocytosis of Ca_V_1.2. Interestingly, the tumor suppressor eIF3e was shown to be responsible for a Ca^2+^-induced internalization of Ca_V_1.2 [151]. It was then suggested that the displacement of α-actinin from the Ca_V_1.2 C-terminus could induce conformational changes that would allow eIF3e to bind to the intracellular II–III loop of Ca_V_1.2 and then trigger its endocytosis [149]. It is also worth noting the presence of a putative AP2 binding site upstream of the IQ motif in Ca_V_1.2 C-terminus that can be unmasked when Ca^2+^ binds to apo-CaM [72].

The stromal interaction molecule 1 (STIM-1), the main activator of store-operated Ca^2+^ channels, was shown to directly interact with the C-terminus of Ca_V_1.2 and reduce its plasma membrane density [152,153]. In hippocampal neurons, STIM-1 affects the depolarization-induced opening of Ca_V_1.2 by both acutely inhibiting its gating and increasing its endocytosis via a dynamin-dependent mechanism [153]. The interaction STIM-1/Ca_V_1.2 was recently investigated in the context of synaptic plasticity in dendritic spines of hippocampal neurons [154]. In this study, the authors showed that the depolarization induced by a brief application of glutamate (15 s) triggers a STIM-1 dependent inhibition of L-type current amplitude. However, this inhibition of L-type current did not involve internalization of the channel as blockers of endocytosis, such as Dyngo4a and Pitstop, did not prevent the reduction in L-type current amplitude. Altogether, these studies suggest that STIM-1 can control Ca_V_1.2 channel activity by different mechanisms depending on the intensity of the stimulus: for brief stimulation, STIM-1 reduces Ca_V_1.2 activity, and for sustained stimuli, STIM-1 induces the internalization of the channels. Thus, STIM-1 provides an important negative feedback mechanism for Ca^2+^ influx.

As noted above, Ca_V_1.2 and Ca_V_2.2 channel surface expression is modulated by ubiquitination, leading to proteasomal degradation. Ca_V_3.2 surface expression is also dependent on ubiquitination, with de-ubiquitinated channels being more stable at the plasma membrane [155]. Two ubiquitin ligases, WWP1 and WWP2, expressed at the cell surface and USP5, a de-ubiquitinase, are critical for the balance ubiquitination/de-ubiquitination of two motifs in the intracellular III–IV linker of Ca_V_3.2. Interestingly, USP5 is up-regulated in animal models of chronic pain and this up-regulation has been linked to the increase in Ca_V_3.2 channel activity and its pro-nociceptive effect [155]. The exact mechanism of how the balance ubiquitination/de-ubiquitination of Ca_V_3.2 channels affect their surface expression is still to be unravelled. However, based on how Nedd4, an E3 ligase that belongs to the same family as WWP1 and WWP2 regulates the epithelial sodium channel ENaC [156], it is likely that the regulation of Ca_V_3.2 involves an endocytic mechanism. It is worth noting that the ubiquitination state of Ca_V_3.2 is modulated by the reversible post-translational addition of small ubiquitin-related modifier (SUMO) peptide on USP5 [157]. Indeed, it has been shown that USP5 SUMOylation decreases Ca_V_3.2/USP5 interaction affinity and then favors Ca_V_3.2 ubiquitination and its degradation.

The collapsin response mediator protein 2 (CRMP2) has been shown to interact with the intracellular I–II loop and C-terminus of Ca_V_2.2 and to increase its cell surface expression [158,159]. The mechanism of action of CRMP2 on Ca_V_2.2 has not yet been fully identified, but it may prevent Ca_V_2.2 endocytosis as it does with Na_v_1.7 channels [160–163]. The effect of CRMP2 on Ca_V_2.2 plasma membrane stability is modulated by post-translational modifications of CRMP2 like phosphorylation and SUMOylation [164,165].

The Ca^2+^ channel and chemotaxis receptor (cache) domain containing 1 protein (Cachd1), was shown to increase Ca_V_2.2 cell surface expression in N2a cells and in hippocampal neurons [29]. Cachd1 protein was identified as an α_2_-δ like protein based on its structural homologies with α_2_-δs: it contains two Cache domains and a VWA domain although with a non-conserved MIDAS motif [166,167]. As opposed to α_2_-δ proteins which affect the forward trafficking of the channel, Cachd1 modifies Ca_V_2.2 trafficking by reducing the rate of endocytosis of the channels [29]. Cachd1 protein was also shown to increase Ca_V_3.1 surface expression in HEK cells and to induce a T-type mediated increase in cell excitability in hippocampal neurons [168]. However, the mechanism by which Cachd1 modulates the trafficking of Ca_V_3.X channels was not investigated [168].

Functional and proteomic analyses of neuronal membranes have revealed a close proximity between Ca_V_2.X channels and voltage- and Ca^2+^-activated potassium (BK) channels [169,170]. More recently, it was demonstrated that BK channels could directly interact with the auxiliary α_2_δ-1 subunit and reduce Ca_V_2.2 plasma membrane trafficking [30]. The co-expression of BK channels with Ca_V_2.2/α_2_δ-1 induces the accumulation of Ca_V_2.2 channels in Rab7-positive intracellular vesicles, a marker for late endosomes and lysosomes, suggesting that BK channels increase the internalization of Ca_V_2.2 channels [30]. These results also suggest that the interaction with BK channels occurs only when the Ca_V_2.2/α_2_δ-1 complex has reached the plasma membrane. Furthermore, the fact that BK channels outcompete Ca_V_2.2 for the binding of α_2_δ-1 and increase Ca_V_2.2 endocytosis is in favor of the idea that α_2_δ-1 has to remain associated with Ca_V_2.2 for the channel complex to stay stably expressed at the plasma membrane [51].

Once they have been endocytosed, channels are targeted either for recycling or for degradation (Figure 4). Very few studies have focused on the pathways involved in the recycling of Ca_V_s. The auxiliary α_2_δ-2 subunit has been shown to be recycled by a Rab11-dependent pathway which controls Ca_V_2.1 current density in tsA-201 cells [171]. In the cardiomyocyte cell line HL-1, Ca_V_1.2 plasma membrane expression was shown to be dependent on the recycling of the channel via a Rab11a-dependent pathway [172]. However, in arterial smooth muscle cells, a Rab25-dependent pathway was shown to be involved in the recycling of Ca_V_1.2 channels [173]. Altogether, these studies suggest a cell type-specific mechanism and further investigation will be needed to identify the pathways involved in the recycling of Ca_V_1.X channels in neurons.

The neuronal actin-binding protein Kelch-like 1 has been identified as a regulator of T-type channel expression [174–177]. In heterologous expression systems, Kelch-like 1 was shown to increase Ca_V_3.2 cell surface targeting in an actin F-dependent manner [177]. The Kelch-like 1 effect on T-type channels was prevented by the co-expression of the dominant negative Rab11-S25N, suggesting the involvement of a Rab11-dependent recycling endosomal pathway [177]. Interestingly, a Rab11-dependent pathway also appears to be involved in the up-regulation of Ca_V_3.2 channel expression by homocysteine [178]. This latter effect on the recycling of Ca_V_3.2 channels relies on the phosphorylation of serine residues located in intracellular domains (loop I–II, loop II–III and C-terminus) by protein kinase C [178]. It is noteworthy that although protein phosphorylation affects various aspects of Ca_V_ channel function [4], very few studies have reported effects on the trafficking of the channels.

## Conclusion

The trafficking of Ca_V_ channels is tightly regulated such that channels can be expressed where and when they are physiologically relevant. In this review we focused on mechanisms that control the trafficking of neuronal Ca_V_ channels from the ER to the plasma membrane, their stability at the plasma membrane and their recycling to intracellular compartments (Figures 2-4). While our understanding of the life cycle of Ca_V_ channels has greatly improved, gaps still remain. For example, how Ca_V_ channels are targeted to the trafficking endosomes and conveyed to specific neuronal subcellular locations is still not fully understood. Moreover, Rab11 has been involved in α_2_δ-2 recycling [171] but we have no experimental evidence whether neuronal Ca_V_α_1_ subunits are taken up by the same pathway or one of the many other existing recycling pathways [179,180]. This is a crucial issue as defects in Ca_V_ trafficking have been linked to pathological conditions such as neuropathic pain and ataxia, and deciphering the intricate mechanisms of Ca_V_ trafficking could allow the development of strategies to correct these defects.

## Abbreviations

ABP: α interaction domain binding pocket
AID: α interaction domain
AP1: Adaptor protein 1
Cachd1: Ca^2+^ channel and chemotaxis receptor domain containing 1 protein
cache: Ca^2+^ channel and chemotaxis receptor
CaM: calmodulin
Ca_V_: voltage-gated Ca^2+^
CRMP2: collapsin response mediator protein 2
cryo-EM: cryogenic electron microscopy
DRG: dorsal root ganglion
eiF3E: Eukaryotic translation initiation factor 3 subunit E
ER: endoplasmic reticulum
FMRP: Fragile X mental retardation protein
GHSR: growth hormone secretagogue receptor type 1a
GPCR: G protein-coupled receptor
HVA: high-voltage-activated channel
LRP1: lipoprotein receptor-related protein-1
LVA: low-voltage-activated channel
MIDAS: metal ion-dependent adhesion site
PIP3: Phosphatidylinositol (3,4,5)-trisphosphate
PKA: protein kinase A
RAP: receptor-associated protein
SH3: src homology 3
Stac3: SH3- and cysteine-rich domain
STIM-1: stromal interaction molecule 1
SUMO: small ubiquitin-related modifier
VWA: von Willebrand factor A

## References

[1] Berridge M.J., Lipp P. and Bootman M.D. (2000) The versatility and universality of calcium signalling. Nat. Rev. Mol. Cell Biol. 1, 11–21 10.1038/35036035 11413485

[2] Clapham D.E. (2007) Calcium signaling. Cell 131, 1047–1058 10.1016/j.cell.2007.11.028 18083096

[3] Dolphin A.C. (2012) Calcium channel auxiliary α2δ and β subunits: trafficking and one step beyond. Nat. Rev. Neurosci. 13, 542–555 10.1038/nrn3311 22805911

[4] Zamponi G.W., Striessnig J., Koschak A. and Dolphin A.C. (2015) The physiology, pathology, and pharmacology of voltage-gated calcium channels and their future therapeutic potential. Pharmacol. Rev. 67, 821–870 10.1124/pr.114.009654 26362469 PMC4630564

[5] Catterall W.A., Lenaeus M.J. and Gamal El-Din T.M. (2020) Structure and pharmacology of voltage-gated sodium and calcium channels. Annu. Rev. Pharmacol. Toxicol. 60, 133–154 10.1146/annurev-pharmtox-010818-021757 31537174

[6] Tang L. et al. (2014) Structural basis for Ca^2+^ selectivity of a voltage-gated calcium channel. Nature 505, 56–61 10.1038/nature12775 24270805 PMC3877713

[7] Wu J. et al. (2016) Structure of the voltage-gated calcium channel Ca(v)1.1 at 3.6 Å resolution. Nature 537, 191–196 10.1038/nature19321 27580036

[8] Buraei Z. and Yang J. (2010) The ß subunit of voltage-gated Ca^2+^ channels. Physiol. Rev. 90, 1461–1506 10.1152/physrev.00057.2009 20959621 PMC4353500

[9] De Waard M., Pragnell M. and Campbell K.P. (1994) Ca^2+^ channel regulation by a conserved beta subunit domain. Neuron 13, 495–503 10.1016/0896-6273(94)90363-8 8060623

[10] Chien A.J. et al. (1995) Roles of a membrane-localized beta subunit in the formation and targeting of functional L-type Ca^2+^ channels. J. Biol. Chem. 270, 30036–30044 10.1074/jbc.270.50.30036 8530407

[11] Yasuda T. et al. (2004) Auxiliary subunit regulation of high-voltage activated calcium channels expressed in mammalian cells. Eur. J. Neurosci. 20, 1–13 10.1111/j.1460-9568.2004.03434.x 15245474

[12] Bichet D. et al. (2000) The I-II loop of the Ca^2+^ channel alpha1 subunit contains an endoplasmic reticulum retention signal antagonized by the beta subunit. Neuron 25, 177–190 10.1016/S0896-6273(00)80881-8 10707982

[13] Gao T., Bunemann M., Gerhardstein B.L., Ma H. and Hosey M.M. (2000) Role of the C terminus of the alpha 1C (CaV1.2) subunit in membrane targeting of cardiac L-type calcium channels. J. Biol. Chem. 275, 25436–25444 10.1074/jbc.M003465200 10816591

[14] Chien A.J., Carr K.M., Shirokov R.E., Rios E. and Hosey M.M. (1996) Identification of palmitoylation sites within the L-type calcium channel beta2a subunit and effects on channel function. J. Biol. Chem. 271, 26465–26468 10.1074/jbc.271.43.26465 8900112

[15] Buraei Z. and Yang J. (2013) Structure and function of the β subunit of voltage-gated Ca^2^ channels. Biochim. Biophys. Acta 1828, 1530–1540 10.1016/j.bbamem.2012.08.028 22981275 PMC3587009

[16] Chen Y.H. et al. (2004) Structural basis of the alpha1-beta subunit interaction of voltage-gated Ca^2+^ channels. Nature 429, 675–680 10.1038/nature02641 15170217

[17] De Waard M., Witcher D.R., Pragnell M., Liu H. and Campbell K.P. (1995) Properties of the alpha 1-beta anchoring site in voltage-dependent Ca^2+^ channels. J. Biol. Chem. 270, 12056–12064 10.1074/jbc.270.20.12056 7744854

[18] Pragnell M. et al. (1994) Calcium channel beta-subunit binds to a conserved motif in the I-II cytoplasmic linker of the alpha 1-subunit. Nature 368, 67–70 10.1038/368067a0 7509046

[19] Maltez J.M., Nunziato D.A., Kim J. and Pitt G.S. (2005) Essential Ca(V)beta modulatory properties are AID-independent. Nat. Struct. Mol. Biol. 12, 372–377 10.1038/nsmb909 15750602

[20] Obermair G.J. et al. (2010) Reciprocal interactions regulate targeting of calcium channel beta subunits and membrane expression of alpha1 subunits in cultured hippocampal neurons. J. Biol. Chem. 285, 5776–5791 10.1074/jbc.M109.044271 19996312 PMC2820804

[21] Nieto-Rostro M., Ramgoolam K., Pratt W.S., Kulik A. and Dolphin A.C. (2018) Ablation of α _2_ δ-1 inhibits cell-surface trafficking of endogenous N-type calcium channels in the pain pathway in vivo. Proc. Natl. Acad Sci. U.S.A. 115, E12043–E12052 10.1073/pnas.1811212115 30487217 PMC6305000

[22] Dolphin A.C. (2018) Voltage-gated calcium channel α_2_δ subunits: an assessment of proposed novel roles. F1000Res. 7, 10.12688/f1000research.16104.1 30519455 PMC6249638

[23] Jay S.D. et al. (1991) Structural characterization of the dihydropyridine-sensitive calcium channel alpha 2-subunit and the associated delta peptides. J. Biol. Chem. 266, 3287–3293 10.1016/S0021-9258(18)49986-3 1847144

[24] De Jongh K.S., Warner C. and Catterall W.A. (1990) Subunits of purified calcium channels. Alpha 2 and delta are encoded by the same gene. J. Biol. Chem. 265, 14738–14741 10.1016/S0021-9258(18)77174-3 2168391

[25] Alvarez-Laviada A., Kadurin I., Senatore A., Chiesa R. and Dolphin A.C. (2014) The inhibition of functional expression of calcium channels by prion protein demonstrates competition with α2δ for GPI-anchoring pathways. Biochem. J. 458, 365–374 10.1042/BJ20131405 24329154 PMC3924758

[26] Kadurin I. et al. (2012) Calcium currents are enhanced by α2δ-1 lacking its membrane anchor. J. Biol. Chem. 287, 33554–33566 10.1074/jbc.M112.378554 22869375 PMC3460456

[27] Davies A. et al. (2010) The alpha2delta subunits of voltage-gated calcium channels form GPI-anchored proteins, a posttranslational modification essential for function. Proc. Natl. Acad Sci. U.S.A. 107, 1654–1659 10.1073/pnas.0908735107 20080692 PMC2824380

[28] Cantí C. et al. (2005) The metal-ion-dependent adhesion site in the Von Willebrand factor-A domain of alpha2delta subunits is key to trafficking voltage-gated Ca2+ channels. Proc. Natl. Acad Sci. U.S.A. 102, 11230–11235 10.1073/pnas.0504183102 16061813 PMC1183569

[29] Dahimene S. et al. (2018) The α _2_ δ-like protein cachd1 increases N-type calcium currents and cell surface expression and competes with α 2 δ-1. Cell Rep. 25, 1610.e1615–1621.e1615 10.1016/j.celrep.2018.10.033 30404013 PMC6231325

[30] Zhang F.X., Gadotti V.M., Souza I.A., Chen L. and Zamponi G.W. (2018) BK potassium channels suppress Cavα2δ subunit function to reduce inflammatory and neuropathic pain. Cell Rep. 22, 1956–1964 10.1016/j.celrep.2018.01.073 29466724

[31] Roth J. et al. (2010) Protein N-glycosylation, protein folding, and protein quality control. Mol. Cells 30, 497–506 10.1007/s10059-010-0159-z 21340671

[32] Moremen K.W., Tiemeyer M. and Nairn A.V. (2012) Vertebrate protein glycosylation: diversity, synthesis and function. Nat. Rev. Mol. Cell Biol. 13, 448–462 10.1038/nrm3383 22722607 PMC3934011

[33] Zhang X. and Wang Y. (2016) Glycosylation quality control by the Golgi structure. J. Mol. Biol. 428, 3183–3193 10.1016/j.jmb.2016.02.030 26956395 PMC4983240

[34] Isaev D. et al. (2007) Role of extracellular sialic acid in regulation of neuronal and network excitability in the rat hippocampus. J. Neurosci. 27, 11587–11594 10.1523/JNEUROSCI.2033-07.2007 17959801 PMC6673228

[35] Montpetit M.L. et al. (2009) Regulated and aberrant glycosylation modulate cardiac electrical signaling. Proc. Natl. Acad Sci. U.S.A. 106, 16517–16522 10.1073/pnas.0905414106 19666501 PMC2752533

[36] Ufret-Vincenty C.A. et al. (2001) Role of sodium channel deglycosylation in the genesis of cardiac arrhythmias in heart failure. J. Biol. Chem. 276, 28197–28203 10.1074/jbc.M102548200 11369778

[37] Watanabe I. et al. (2003) Glycosylation affects rat Kv1.1 potassium channel gating by a combined surface potential and cooperative subunit interaction mechanism. J. Physiol. 550, 51–66 10.1113/jphysiol.2003.040337 12879861 PMC2343013

[38] Watanabe I., Zhu J., Recio-Pinto E. and Thornhill W.B. (2004) Glycosylation affects the protein stability and cell surface expression of Kv1.4 but Not Kv1.1 potassium channels. A pore region determinant dictates the effect of glycosylation on trafficking. J. Biol. Chem. 279, 8879–8885 10.1074/jbc.M309802200 14688283

[39] Dietrich A. et al. (2003) N-linked protein glycosylation is a major determinant for basal TRPC3 and TRPC6 channel activity. J. Biol. Chem. 278, 47842–47852 10.1074/jbc.M302983200 12970363

[40] Pertusa M., Madrid R., Morenilla-Palao C., Belmonte C. and Viana F. (2012) N-glycosylation of TRPM8 ion channels modulates temperature sensitivity of cold thermoreceptor neurons. J. Biol. Chem. 287, 18218–18229 10.1074/jbc.M111.312645 22493431 PMC3365712

[41] Xu H., Fu Y., Tian W. and Cohen D.M. (2006) Glycosylation of the osmoresponsive transient receptor potential channel TRPV4 on Asn-651 influences membrane trafficking. Am. J. Physiol. Renal Physiol. 290, F1103–F1109 10.1152/ajprenal.00245.2005 16368742

[42] Ficelova V. et al. (2020) Functional identification of potential non-canonical N-glycosylation sites within Cav3.2 T-type calcium channels. Mol. Brain 13, 149 10.1186/s13041-020-00697-z 33176830 PMC7659234

[43] Lazniewska J. and Weiss N. (2017) Glycosylation of voltage-gated calcium channels in health and disease. Biochim. Biophys. Acta Biomembr. 1859, 662–668 10.1016/j.bbamem.2017.01.018 28109749

[44] Weiss N., Black S.A., Bladen C., Chen L. and Zamponi G.W. (2013) Surface expression and function of Cav3.2 T-type calcium channels are controlled by asparagine-linked glycosylation. Pflugers Arch. 465, 1159–1170 10.1007/s00424-013-1259-3 23503728

[45] Orestes P. et al. (2013) Reversal of neuropathic pain in diabetes by targeting glycosylation of Ca(V)3.2 T-type calcium channels. Diabetes 62, 3828–3838 10.2337/db13-0813 23835327 PMC3806612

[46] Ondacova K., Karmazinova M., Lazniewska J., Weiss N. and Lacinova L. (2016) Modulation of Cav3.2 T-type calcium channel permeability by asparagine-linked glycosylation. Channels (Austin) 10, 175–184 10.1080/19336950.2016.1138189 26745591 PMC4954584

[47] Lazniewska J., Rzhepetskyy Y., Zhang F.X., Zamponi G.W. and Weiss N. (2016) Cooperative roles of glucose and asparagine-linked glycosylation in T-type calcium channel expression. Pflugers Arch. 468, 1837–1851 10.1007/s00424-016-1881-y 27659162

[48] Weiss N. and Zamponi G.W. (2019) T-type calcium channels: from molecule to therapeutic opportunities. Int. J. Biochem. Cell Biol. 108, 34–39 10.1016/j.biocel.2019.01.008 30648620

[49] Joksimovic S.L. et al. (2020) Glycosylation of Ca_V_3.2 channels contributes to the hyperalgesia in peripheral neuropathy of type 1 diabetes. Front. Cell. Neurosci. 14, 605312 10.3389/fncel.2020.605312 33384586 PMC7770106

[50] Zamponi G.W. (2016) Targeting voltage-gated calcium channels in neurological and psychiatric diseases. Nat. Rev. Drug Discov. 15, 19–34 10.1038/nrd.2015.5 26542451

[51] Cassidy J.S., Ferron L., Kadurin I., Pratt W.S. and Dolphin A.C. (2014) Functional exofacially tagged N-type calcium channels elucidate the interaction with auxiliary α2δ-1 subunits. Proc. Natl. Acad Sci. U.S.A. 111, 8979–8984 10.1073/pnas.1403731111 24889613 PMC4066493

[52] Marais E., Klugbauer N. and Hofmann F. (2001) Calcium channel alpha(2)delta subunits-structure and Gabapentin binding. Mol. Pharmacol. 59, 1243–1248 10.1124/mol.59.5.1243 11306709

[53] Tétreault M.P. et al. (2016) Identification of glycosylation sites essential for surface expression of the CaVα2δ1 subunit and modulation of the cardiac CaV1.2 channel activity. J. Biol. Chem. 291, 4826–4843 10.1074/jbc.M115.692178 26742847 PMC4813503

[54] Sandoval A., Oviedo N., Andrade A. and Felix R. (2004) Glycosylation of asparagines 136 and 184 is necessary for the alpha2delta subunit-mediated regulation of voltage-gated Ca^2+^ channels. FEBS Lett. 576, 21–26 10.1016/j.febslet.2004.08.054 15474003

[55] Kadurin I., Rothwell S.W., Lana B., Nieto-Rostro M. and Dolphin A.C. (2017) LRP1 influences trafficking of N-type calcium channels via interaction with the auxiliary α_2_δ-1 subunit. Sci. Rep. 7, 43802 10.1038/srep43802 28256585 PMC5335561

[56] Dolphin A.C. (2013) The α2δ subunits of voltage-gated calcium channels. Biochim. Biophys. Acta 1828, 1541–1549 10.1016/j.bbamem.2012.11.019 23196350

[57] Ferron L., Kadurin I. and Dolphin A.C. (2018) Proteolytic maturation of α2δ controls the probability of synaptic vesicular release. Elife 7, e37507 10.7554/eLife.37507 29916807 PMC6029843

[58] Kadurin I. et al. (2016) Proteolytic maturation of α_2_δ represents a checkpoint for activation and neuronal trafficking of latent calcium channels. Elife 5, e21143 10.7554/eLife.21143 27782881 PMC5092059

[59] Cornet V. et al. (2002) Multiple determinants in voltage-dependent P/Q calcium channels control their retention in the endoplasmic reticulum. Eur. J. Neurosci. 16, 883–895 10.1046/j.1460-9568.2002.02168.x 12372025

[60] Altier C. et al. (2011) The Cavβ subunit prevents RFP2-mediated ubiquitination and proteasomal degradation of L-type channels. Nat. Neurosci. 14, 173–180 10.1038/nn.2712 21186355

[61] Fang K. and Colecraft H.M. (2011) Mechanism of auxiliary β-subunit-mediated membrane targeting of L-type (Ca(V)1.2) channels. J. Physiol. 589, 4437–4455 10.1113/jphysiol.2011.214247 21746784 PMC3208217

[62] Arias J.M., Murbartián J., Vitko I., Lee J.H. and Perez-Reyes E. (2005) Transfer of beta subunit regulation from high to low voltage-gated Ca^2+^ channels. FEBS Lett. 579, 3907–3912 10.1016/j.febslet.2005.06.008 15987636

[63] Waithe D., Ferron L., Page K.M., Chaggar K. and Dolphin A.C. (2011) Beta-subunits promote the expression of Ca(V)2.2 channels by reducing their proteasomal degradation. J. Biol. Chem. 286, 9598–9611 10.1074/jbc.M110.195909 21233207 PMC3059031

[64] Leroy J. et al. (2005) Interaction via a key tryptophan in the I-II linker of N-type calcium channels is required for beta1 but not for palmitoylated beta2, implicating an additional binding site in the regulation of channel voltage-dependent properties. J. Neurosci. 25, 6984–6996 10.1523/JNEUROSCI.1137-05.2005 16049174 PMC6724838

[65] Page K.M., Rothwell S.W. and Dolphin A.C. (2016) The CaVβ subunit protects the I-II loop of the voltage-gated calcium channel CaV2.2 from proteasomal degradation but not oligoubiquitination. J. Biol. Chem. 291, 20402–20416 10.1074/jbc.M116.737270 27489103 PMC5034038

[66] Rougier J.S., Albesa M., Abriel H. and Viard P. (2011) Neuronal precursor cell-expressed developmentally down-regulated 4-1 (NEDD4-1) controls the sorting of newly synthesized Ca(V)1.2 calcium channels. J. Biol. Chem. 286, 8829–8838 10.1074/jbc.M110.166520 21220429 PMC3059038

[67] Viard P. et al. (2004) PI3K promotes voltage-dependent calcium channel trafficking to the plasma membrane. Nat. Neurosci. 7, 939–946 10.1038/nn1300 15311280

[68] Catalucci D. et al. (2009) Akt regulates L-type Ca^2+^ channel activity by modulating Cavalpha1 protein stability. J. Cell Biol. 184, 923–933 10.1083/jcb.200805063 19307602 PMC2699149

[69] López Soto E.J. et al. (2015) Constitutive and ghrelin-dependent GHSR1a activation impairs CaV2.1 and CaV2.2 currents in hypothalamic neurons. J. Gen. Physiol. 146, 205–219 10.1085/jgp.201511383 26283199 PMC4555474

[70] Mustafá E.R. et al. (2017) Constitutive activity of the Ghrelin receptor reduces surface expression of voltage-gated Ca^2+^ channels in a Ca_V_β-dependent manner. J. Cell Sci. 130, 3907–3917 10.1242/jcs.207886 29038230 PMC6518300

[71] Bonifacino J.S. (2014) Adaptor proteins involved in polarized sorting. J. Cell Biol. 204, 7–17 10.1083/jcb.201310021 24395635 PMC3882786

[72] Macabuag N. and Dolphin A.C. (2015) Alternative splicing in Ca(V)2.2 regulates neuronal trafficking via adaptor protein complex-1 adaptor protein motifs. J. Neurosci. 35, 14636–14652 10.1523/JNEUROSCI.3034-15.2015 26511252 PMC4623230

[73] Bell T.J., Thaler C., Castiglioni A.J., Helton T.D. and Lipscombe D. (2004) Cell-specific alternative splicing increases calcium channel current density in the pain pathway. Neuron 41, 127–138 10.1016/S0896-6273(03)00801-8 14715140

[74] Lipscombe D., Pan J.Q. and Gray A.C. (2002) Functional diversity in neuronal voltage-gated calcium channels by alternative splicing of Ca(v)alpha1. Mol. Neurobiol. 26, 21–44 10.1385/MN:26:1:021 12392054

[75] Altier C. et al. (2007) Differential role of N-type calcium channel splice isoforms in pain. J. Neurosci. 27, 6363–6373 10.1523/JNEUROSCI.0307-07.2007 17567797 PMC6672448

[76] Contractor A., Klyachko V.A. and Portera-Cailliau C. (2015) Altered neuronal and circuit excitability in Fragile X syndrome. Neuron 87, 699–715 10.1016/j.neuron.2015.06.017 26291156 PMC4545495

[77] Zhan X. et al. (2020) FMRP(1-297)-tat restores ion channel and synaptic function in a model of Fragile X syndrome. Nat. Commun. 11, 2755 10.1038/s41467-020-16250-4 32488011 PMC7265297

[78] Ferron L. (2016) Fragile X mental retardation protein controls ion channel expression and activity. J. Physiol. 594, 5861–5867 10.1113/JP270675 26864773 PMC5063927

[79] Ferron L., Nieto-Rostro M., Cassidy J.S. and Dolphin A.C. (2014) Fragile X mental retardation protein controls synaptic vesicle exocytosis by modulating N-type calcium channel density. Nat. Commun. 5, 3628 10.1038/ncomms4628 24709664 PMC3982139

[80] Ferron L. et al. (2020) FMRP regulates presynaptic localization of neuronal voltage gated calcium channels. Neurobiol. Dis. 138, 104779 10.1016/j.nbd.2020.104779 31991246 PMC7152798

[81] Nelson B.R. et al. (2013) Skeletal muscle-specific T-tubule protein STAC3 mediates voltage-induced Ca^2+^ release and contractility. Proc. Natl. Acad Sci. U.S.A. 110, 11881–11886 10.1073/pnas.1310571110 23818578 PMC3718085

[82] Horstick E.J. et al. (2013) Stac3 is a component of the excitation-contraction coupling machinery and mutated in Native American myopathy. Nat. Commun. 4, 1952 10.1038/ncomms2952 23736855 PMC4056023

[83] Polster A., Perni S., Bichraoui H. and Beam K.G. (2015) Stac adaptor proteins regulate trafficking and function of muscle and neuronal L-type Ca^2+^ channels. Proc. Natl. Acad Sci. U.S.A. 112, 602–606 10.1073/pnas.1423113112 25548159 PMC4299259

[84] Campiglio M. et al. (2018) STAC proteins associate to the IQ domain of Ca_V_1.2 and inhibit calcium-dependent inactivation. Proc. Natl. Acad Sci. U.S.A. 115, 1376–1381 10.1073/pnas.1715997115 29363593 PMC5819422

[85] Polster A. et al. (2018) Stac proteins suppress Ca^2+^-dependent inactivation of neuronal l-type Ca^2+^ channels. J. Neurosci. 38, 9215–9227 10.1523/JNEUROSCI.0695-18.2018 30201773 PMC6199411

[86] Rzhepetskyy Y. et al. (2016) A Cav3.2/Stac1 molecular complex controls T-type channel expression at the plasma membrane. Channels (Austin) 10, 346–354 10.1080/19336950.2016.1186318 27149520 PMC4988463

[87] Snutch T.P., Tomlinson W.J., Leonard J.P. and Gilbert M.M. (1991) Distinct calcium channels are generated by alternative splicing and are differentially expressed in the mammalian CNS. Neuron 7, 45–57 10.1016/0896-6273(91)90073-9 1648941

[88] Bourinet E. et al. (1999) Splicing of alpha 1A subunit gene generates phenotypic variants of P- and Q-type calcium channels. Nat. Neurosci. 2, 407–415 10.1038/8070 10321243

[89] Lin Z., Haus S., Edgerton J. and Lipscombe D. (1997) Identification of functionally distinct isoforms of the N-type Ca^2+^ channel in rat sympathetic ganglia and brain. Neuron 18, 153–166 10.1016/S0896-6273(01)80054-4 9010213

[90] Gray A.C., Raingo J. and Lipscombe D. (2007) Neuronal calcium channels: splicing for optimal performance. Cell Calcium 42, 409–417 10.1016/j.ceca.2007.04.003 17512586 PMC2001240

[91] Lipscombe D., Andrade A. and Allen S.E. (2013) Alternative splicing: functional diversity among voltage-gated calcium channels and behavioral consequences. Biochim. Biophys. Acta 1828, 1522–1529 10.1016/j.bbamem.2012.09.018 23022282 PMC3625486

[92] Perez-Reyes E. (2003) Molecular physiology of low-voltage-activated t-type calcium channels. Physiol. Rev. 83, 117–161 10.1152/physrev.00018.2002 12506128

[93] Ahern C.A. et al. (2001) Intramembrane charge movements and excitation- contraction coupling expressed by two-domain fragments of the Ca^2+^ channel. Proc. Natl. Acad Sci. U.S.A. 98, 6935–6940 10.1073/pnas.111001898 11371610 PMC34456

[94] Wielowieyski P.A., Wigle J.T., Salih M., Hum P. and Tuana B.S. (2001) Alternative splicing in intracellular loop connecting domains II and III of the alpha 1 subunit of Cav1.2 Ca^2+^ channels predicts two-domain polypeptides with unique C-terminal tails. J. Biol. Chem. 276, 1398–1406 10.1074/jbc.M006868200 11010971

[95] Arikkath J. et al. (2002) Molecular characterization of a two-domain form of the neuronal voltage-gated P/Q-type calcium channel alpha(1)2.1 subunit. FEBS Lett. 532, 300–308 10.1016/S0014-5793(02)03693-1 12482583

[96] Okagaki R. et al. (2001) The maternal transcript for truncated voltage-dependent Ca^2+^ channels in the ascidian embryo: a potential suppressive role in Ca^2+^ channel expression. Dev. Biol. 230, 258–277 10.1006/dbio.2000.0119 11161577

[97] Ophoff R.A. et al. (1996) Familial hemiplegic migraine and episodic ataxia type-2 are caused by mutations in the Ca^2+^ channel gene CACNL1A4. Cell 87, 543–552 10.1016/S0092-8674(00)81373-2 8898206

[98] Jouvenceau A. et al. (2001) Human epilepsy associated with dysfunction of the brain P/Q-type calcium channel. Lancet 358, 801–807 10.1016/S0140-6736(01)05971-2 11564488

[99] Page K.M. et al. (2004) Dominant-negative calcium channel suppression by truncated constructs involves a kinase implicated in the unfolded protein response. J. Neurosci. 24, 5400–5409 10.1523/JNEUROSCI.0553-04.2004 15190113 PMC6729303

[100] Raghib A. et al. (2001) Dominant-negative synthesis suppression of voltage-gated calcium channel Cav2.2 induced by truncated constructs. J. Neurosci. 21, 8495–8504 10.1523/JNEUROSCI.21-21-08495.2001 11606638 PMC6762802

[101] Mezghrani A. et al. (2008) A destructive interaction mechanism accounts for dominant-negative effects of misfolded mutants of voltage-gated calcium channels. J. Neurosci. 28, 4501–4511 10.1523/JNEUROSCI.2844-07.2008 18434528 PMC6670939

[102] Dahimene S. et al. (2016) A CaV2.1 N-terminal fragment relieves the dominant-negative inhibition by an Episodic ataxia 2 mutant. Neurobiol. Dis. 93, 243–256 10.1016/j.nbd.2016.05.020 27260834 PMC4940211

[103] Page K.M. et al. (2010) N terminus is key to the dominant negative suppression of Ca(V)2 calcium channels: implications for episodic ataxia type 2. J. Biol. Chem. 285, 835–844 10.1074/jbc.M109.065045 19903821 PMC2801285

[104] Meyer J.O. et al. (2019) Disruption of the key Ca^2+^ binding site in the selectivity filter of neuronal voltage-gated calcium channels inhibits channel trafficking. Cell Rep. 29, 22.e25–33.e25 10.1016/j.celrep.2019.08.079 31577951 PMC6899504

[105] Ben-Johny M. and Yue D.T. (2014) Calmodulin regulation (calmodulation) of voltage-gated calcium channels. J. Gen. Physiol. 143, 679–692 10.1085/jgp.201311153 24863929 PMC4035741

[106] Dolmetsch R.E., Pajvani U., Fife K., Spotts J.M. and Greenberg M.E. (2001) Signaling to the nucleus by an L-type calcium channel-calmodulin complex through the MAP kinase pathway. Science 294, 333–339 10.1126/science.1063395 11598293

[107] Bernatchez G., Talwar D. and Parent L. (1998) Mutations in the EF-hand motif impair the inactivation of barium currents of the cardiac alpha1C channel. Biophys. J. 75, 1727–1739 10.1016/S0006-3495(98)77614-3 9746514 PMC1299844

[108] Peterson B.Z. et al. (2000) Critical determinants of Ca(2+)-dependent inactivation within an EF-hand motif of L-type Ca(2+) channels. Biophys. J. 78, 1906–1920 10.1016/S0006-3495(00)76739-7 10733970 PMC1300784

[109] Bourdin B. et al. (2010) Molecular determinants of the CaVbeta-induced plasma membrane targeting of the CaV1.2 channel. J. Biol. Chem. 285, 22853–22863 10.1074/jbc.M110.111062 20478999 PMC2906277

[110] Davare M.A. et al. (2001) A beta2 adrenergic receptor signaling complex assembled with the Ca^2+^ channel Cav1.2. Science 293, 98–101 10.1126/science.293.5527.98 11441182

[111] Patriarchi T. et al. (2016) Phosphorylation of Cav1.2 on S1928 uncouples the L-type Ca^2+^ channel from the β2 adrenergic receptor. EMBO J. 35, 1330–1345 10.15252/embj.201593409 27103070 PMC4910527

[112] Kitano J. et al. (2003) Direct interaction and functional coupling between metabotropic glutamate receptor subtype 1 and voltage-sensitive Cav2.1 Ca^2+^ channel. J. Biol. Chem. 278, 25101–25108 10.1074/jbc.M303266200 12704197

[113] Beedle A.M. et al. (2004) Agonist-independent modulation of N-type calcium channels by ORL1 receptors. Nat. Neurosci. 7, 118–125 10.1038/nn1180 14730309

[114] Altier C. et al. (2006) ORL1 receptor-mediated internalization of N-type calcium channels. Nat. Neurosci. 9, 31–40 10.1038/nn1605 16311589

[115] Evans R.M. et al. (2010) Heterodimerization of ORL1 and opioid receptors and its consequences for N-type calcium channel regulation. J. Biol. Chem. 285, 1032–1040 10.1074/jbc.M109.040634 19887453 PMC2801230

[116] Kisilevsky A.E. et al. (2008) D1 receptors physically interact with N-type calcium channels to regulate channel distribution and dendritic calcium entry. Neuron 58, 557–570 10.1016/j.neuron.2008.03.002 18498737

[117] Kisilevsky A.E. and Zamponi G.W. (2008) D2 dopamine receptors interact directly with N-type calcium channels and regulate channel surface expression levels. Channels (Austin) 2, 269–277 10.4161/chan.2.4.6402 18719394

[118] Laviv T. et al. (2011) Compartmentalization of the GABAB receptor signaling complex is required for presynaptic inhibition at hippocampal synapses. J. Neurosci. 31, 12523–12532 10.1523/JNEUROSCI.1527-11.2011 21880914 PMC6703276

[119] Schwenk J. et al. (2016) Modular composition and dynamics of native GABAB receptors identified by high-resolution proteomics. Nat. Neurosci. 19, 233–242 10.1038/nn.4198 26691831

[120] Benleulmi-Chaachoua A. et al. (2016) Protein interactome mining defines melatonin MT1 receptors as integral component of presynaptic protein complexes of neurons. J. Pineal Res. 60, 95–108 10.1111/jpi.12294 26514267

[121] Tedford H.W. and Zamponi G.W. (2006) Direct G protein modulation of Cav2 calcium channels. Pharmacol. Rev. 58, 837–862 10.1124/pr.58.4.11 17132857

[122] Zamponi G.W. (2001) Determinants of G protein inhibition of presynaptic calcium channels. Cell Biochem. Biophys. 34, 79–94 10.1385/CBB:34:1:79 11394442

[123] Zamponi G.W. and Currie K.P. (2013) Regulation of Ca(V)2 calcium channels by G protein coupled receptors. Biochim. Biophys. Acta 1828, 1629–1643 10.1016/j.bbamem.2012.10.004 23063655 PMC3556207

[124] Dolphin A.C. (2003) G protein modulation of voltage-gated calcium channels. Pharmacol. Rev. 55, 607–627 10.1124/pr.55.4.3 14657419

[125] Gray R. and Johnston D. (1987) Noradrenaline and beta-adrenoceptor agonists increase activity of voltage-dependent calcium channels in hippocampal neurons. Nature 327, 620–622 10.1038/327620a0 2439913

[126] Zamponi G.W., Bourinet E., Nelson D., Nargeot J. and Snutch T.P. (1997) Crosstalk between G proteins and protein kinase C mediated by the calcium channel alpha1 subunit. Nature 385, 442–446 10.1038/385442a0 9009192

[127] De Waard M. et al. (1997) Direct binding of G-protein betagamma complex to voltage-dependent calcium channels. Nature 385, 446–450 10.1038/385446a0 9009193

[128] DePuy S.D. et al. (2006) The molecular basis for T-type Ca^2+^ channel inhibition by G protein beta2gamma2 subunits. Proc. Natl. Acad Sci. U.S.A. 103, 14590–14595 10.1073/pnas.0603945103 16973746 PMC1600004

[129] Wolfe J.T., Wang H., Howard J., Garrison J.C. and Barrett P.Q. (2003) T-type calcium channel regulation by specific G-protein betagamma subunits. Nature 424, 209–213 10.1038/nature01772 12853961

[130] Wang H.L. et al. (2005) Heterodimerization of opioid receptor-like 1 and mu-opioid receptors impairs the potency of micro receptor agonist. J. Neurochem. 92, 1285–1294 10.1111/j.1471-4159.2004.02921.x 15748148

[131] Reynet C. and Kahn C.R. (1993) Rad: a member of the Ras family overexpressed in muscle of type II diabetic humans. Science 262, 1441–1444 10.1126/science.8248782 8248782

[132] Maguire J. et al. (1994) Gem: an induced, immediate early protein belonging to the Ras family. Science 265, 241–244 10.1126/science.7912851 7912851

[133] Finlin B.S. and Andres D.A. (1997) Rem is a new member of the Rad- and Gem/Kir Ras-related GTP-binding protein family repressed by lipopolysaccharide stimulation. J. Biol. Chem. 272, 21982–21988 10.1074/jbc.272.35.21982 9268335

[134] Finlin B.S., Shao H., Kadono-Okuda K., Guo N. and Andres D.A. (2000) Rem2, a new member of the Rem/Rad/Gem/Kir family of Ras-related GTPases. Biochem. J. 347, 223–231 10.1042/bj3470223 10727423 PMC1220952

[135] Yang T. and Colecraft H.M. (2013) Regulation of voltage-dependent calcium channels by RGK proteins. Biochim. Biophys. Acta 1828, 1644–1654 10.1016/j.bbamem.2012.10.005 23063948 PMC4190112

[136] Yang T., Xu X., Kernan T., Wu V. and Colecraft H.M. (2010) Rem, a member of the RGK GTPases, inhibits recombinant CaV1.2 channels using multiple mechanisms that require distinct conformations of the GTPase. J. Physiol. 588, 1665–1681 10.1113/jphysiol.2010.187203 20308247 PMC2887986

[137] Béguin P. et al. (2001) Regulation of Ca^2+^ channel expression at the cell surface by the small G-protein kir/Gem. Nature 411, 701–706 10.1038/35079621 11395774

[138] Puckerin A.A., Chang D.D., Subramanyam P. and Colecraft H.M. (2016) Similar molecular determinants on Rem mediate two distinct modes of inhibition of Ca_V_1.2 channels. Channels (Austin) 10, 379–394 10.1080/19336950.2016.1180489 27115600 PMC4988437

[139] Puckerin A.A. et al. (2018) Engineering selectivity into RGK GTPase inhibition of voltage-dependent calcium channels. Proc. Natl. Acad Sci. U.S.A. 115, 12051–12056 10.1073/pnas.1811024115 30397133 PMC6255209

[140] Yang T., Puckerin A. and Colecraft H.M. (2012) Distinct RGK GTPases differentially use α1- and auxiliary β-binding-dependent mechanisms to inhibit CaV1.2/CaV2.2 channels. PLoS ONE 7, e37079 10.1371/journal.pone.0037079 22590648 PMC3349659

[141] Liu G. et al. (2020) Mechanism of adrenergic Ca_V_1.2 stimulation revealed by proximity proteomics. Nature 577, 695–700 10.1038/s41586-020-1947-z 31969708 PMC7018383

[142] Papa A. et al. (2021) Adrenergic Ca_V_1.2 activation via Rad phosphorylation converges at α _1C_ I-II loop. Circ. Res. 128, 76–88 10.1161/CIRCRESAHA.120.317839 33086983 PMC7790865

[143] Cachelin A.B., de Peyer J.E., Kokubun S. and Reuter H. (1983) Ca^2+^ channel modulation by 8-bromocyclic AMP in cultured heart cells. Nature 304, 462–464 10.1038/304462a0 6308462

[144] Catterall W.A. (2015) Regulation of cardiac calcium channels in the fight-or-flight response. Curr. Mol. Pharmacol. 8, 12–21 10.2174/1874467208666150507103417 25966697 PMC4664455

[145] Colecraft H.M. (2020) Designer genetically encoded voltage-dependent calcium channel inhibitors inspired by RGK GTPases. J. Physiol. 598, 1683–1693 10.1113/JP276544 32104913 PMC7195252

[146] Makarewich C.A. et al. (2012) A caveolae-targeted L-type Ca²+ channel antagonist inhibits hypertrophic signaling without reducing cardiac contractility. Circ. Res. 110, 669–674 10.1161/CIRCRESAHA.111.264028 22302787 PMC3324037

[147] Gonzalez-Gutierrez G., Miranda-Laferte E., Neely A. and Hidalgo P. (2007) The Src homology 3 domain of the beta-subunit of voltage-gated calcium channels promotes endocytosis via dynamin interaction. J. Biol. Chem. 282, 2156–2162 10.1074/jbc.M609071200 17110381

[148] Miranda-Laferte E. et al. (2011) Homodimerization of the Src homology 3 domain of the calcium channel β-subunit drives dynamin-dependent endocytosis. J. Biol. Chem. 286, 22203–22210 10.1074/jbc.M110.201871 21502319 PMC3121365

[149] Hall D.D. et al. (2013) Competition between α-actinin and Ca²-calmodulin controls surface retention of the L-type Ca^2^ channel Ca(V)1.2. Neuron 78, 483–497 10.1016/j.neuron.2013.02.032 23664615 PMC4570828

[150] Turner M. et al. (2020) α-Actinin-1 promotes activity of the L-type Ca^2+^ channel Ca_v_1.2. EMBO J. 39, e102622 10.15252/embj.2020106171 33433005 PMC7507692

[151] Green E.M., Barrett C.F., Bultynck G., Shamah S.M. and Dolmetsch R.E. (2007) The tumor suppressor eIF3e mediates calcium-dependent internalization of the L-type calcium channel CaV1.2. Neuron 55, 615–632 10.1016/j.neuron.2007.07.024 17698014 PMC2384234

[152] Wang Y. et al. (2010) The calcium store sensor, STIM1, reciprocally controls Orai and CaV1.2 channels. Science 330, 105–109 10.1126/science.1191086 20929813 PMC3601900

[153] Park C.Y., Shcheglovitov A. and Dolmetsch R. (2010) The CRAC channel activator STIM1 binds and inhibits L-type voltage-gated calcium channels. Science 330, 101–105 10.1126/science.1191027 20929812

[154] Dittmer P.J., Wild A.R., Dell’Acqua M.L. and Sather W.A. (2017) STIM1 Ca^2+^ sensor control of L-type Ca^2+^-channel-dependent dendritic spine structural plasticity and nuclear signaling. Cell Rep. 19, 321–334 10.1016/j.celrep.2017.03.056 28402855 PMC5451256

[155] García-Caballero A. et al. (2014) The deubiquitinating enzyme USP5 modulates neuropathic and inflammatory pain by enhancing Cav3.2 channel activity. Neuron 83, 1144–1158 10.1016/j.neuron.2014.07.036 25189210

[156] Abriel H. and Staub O. (2005) Ubiquitylation of ion channels. Physiology (Bethesda) 20, 398–407 10.1152/physiol.00033.2005 16287989

[157] Garcia-Caballero A. et al. (2019) SUMOylation regulates USP5-Cav3.2 calcium channel interactions. Mol. Brain 12, 73 10.1186/s13041-019-0493-9 31455361 PMC6712834

[158] Chi X.X. et al. (2009) Regulation of N-type voltage-gated calcium channels (Cav2.2) and transmitter release by collapsin response mediator protein-2 (CRMP-2) in sensory neurons. J. Cell Sci. 122, 4351–4362 10.1242/jcs.053280 19903690 PMC2779133

[159] Brittain J.M. et al. (2009) An atypical role for collapsin response mediator protein 2 (CRMP-2) in neurotransmitter release via interaction with presynaptic voltage-gated calcium channels. J. Biol. Chem. 284, 31375–31390 10.1074/jbc.M109.009951 19755421 PMC2781534

[160] Fukata Y. et al. (2002) CRMP-2 binds to tubulin heterodimers to promote microtubule assembly. Nat. Cell Biol. 4, 583–591 10.1038/ncb825 12134159

[161] Rahajeng J., Giridharan S.S., Naslavsky N. and Caplan S. (2010) Collapsin response mediator protein-2 (Crmp2) regulates trafficking by linking endocytic regulatory proteins to dynein motors. J. Biol. Chem. 285, 31918–31922 10.1074/jbc.C110.166066 20801876 PMC2952192

[162] Dustrude E.T. et al. (2016) Hierarchical CRMP2 posttranslational modifications control NaV1.7 function. Proc. Natl. Acad Sci. U.S.A. 113, E8443–E8452 10.1073/pnas.1610531113 27940916 PMC5206544

[163] Moutal A. et al. (2019) Dysregulation of CRMP2 post-translational modifications drive its pathological functions. Mol. Neurobiol. 56, 6736–6755 10.1007/s12035-019-1568-4 30915713 PMC6728212

[164] Moutal A. et al. (2016) (S)-Lacosamide binding to Collapsin Response Mediator Protein 2 (CRMP2) regulates CaV2.2 activity by subverting its phosphorylation by Cdk5. Mol. Neurobiol. 53, 1959–1976 10.1007/s12035-015-9141-2 25846820

[165] Ju W. et al. (2013) SUMOylation alters CRMP2 regulation of calcium influx in sensory neurons. Channels (Austin) 7, 153–159 10.4161/chan.24224 23510938 PMC3710342

[166] Stephens G.J. and Cottrell G.S. (2019) CACHD1: a new activity-modifying protein for voltage-gated calcium channels. Channels (Austin) 13, 120–123 10.1080/19336950.2019.1600968 30983497 PMC6527056

[167] Anantharaman V. and Aravind L. (2000) Cache - a signaling domain common to animal Ca(2+)-channel subunits and a class of prokaryotic chemotaxis receptors. Trends Biochem. Sci. 25, 535–537 10.1016/S0968-0004(00)01672-8 11084361

[168] Cottrell G.S. et al. (2018) CACHD1 is an α2δ-like protein that modulates Ca_V_3 voltage-gated calcium channel activity. J. Neurosci. 38, 9186–9201 10.1523/JNEUROSCI.3572-15.2018 30181139 PMC6199404

[169] Berkefeld H. et al. (2006) BKCa-Cav channel complexes mediate rapid and localized Ca^2+^-activated K+ signaling. Science 314, 615–620 10.1126/science.1132915 17068255

[170] Müller C.S. et al. (2010) Quantitative proteomics of the Cav2 channel nano-environments in the mammalian brain. Proc. Natl. Acad Sci. U.S.A. 107, 14950–14957 10.1073/pnas.1005940107 20668236 PMC2930569

[171] Tran-Van-Minh A. and Dolphin A.C. (2010) The alpha2delta ligand gabapentin inhibits the Rab11-dependent recycling of the calcium channel subunit alpha2delta-2. J. Neurosci. 30, 12856–12867 10.1523/JNEUROSCI.2700-10.2010 20861389 PMC6633565

[172] Conrad R. et al. (2018) Rapid turnover of the cardiac L-type Ca. iScience 7, 1–15 10.1016/j.isci.2018.08.012 30267672 PMC6135870

[173] Bannister J.P., Bulley S., Leo M.D., Kidd M.W. and Jaggar J.H. (2016) Rab25 influences functional Cav1.2 channel surface expression in arterial smooth muscle cells. Am. J. Physiol. Cell Physiol. 310, C885–C893 10.1152/ajpcell.00345.2015 27076616 PMC4935198

[174] Perissinotti P.P. et al. (2014) Calcium current homeostasis and synaptic deficits in hippocampal neurons from Kelch-like 1 knockout mice. Front. Cell. Neurosci. 8, 444 25610372 10.3389/fncel.2014.00444 PMC4285801

[175] Perissinotti P.P. et al. (2014) Down-regulation of endogenous KLHL1 decreases voltage-gated calcium current density. Cell Calcium 55, 269–280 10.1016/j.ceca.2014.03.002 24703904

[176] Aromolaran K.A., Benzow K.A., Cribbs L.L., Koob M.D. and Piedras-Rentería E.S. (2010) T-type current modulation by the actin-binding protein Kelch-like 1. Am. J. Physiol. Cell Physiol. 298, C1353–C1362 10.1152/ajpcell.00235.2009 20147652

[177] Aromolaran K.A., Benzow K.A., Cribbs L.L., Koob M.D. and Piedras-Rentería E.S. (2009) Kelch-like 1 protein upregulates T-type currents by an actin-F dependent increase in α(1H) channels via the recycling endosome. Channels (Austin) 3, 402–412 10.4161/chan.3.6.9858 19806008

[178] Gaifullina A.S. et al. (2019) A potential role for T-type calcium channels in homocysteinemia-induced peripheral neuropathy. Pain 160, 2798–2810 10.1097/j.pain.0000000000001669 31365467

[179] Bhuin T. and Roy J.K. (2014) Rab proteins: the key regulators of intracellular vesicle transport. Exp. Cell. Res. 328, 1–19 10.1016/j.yexcr.2014.07.027 25088255

[180] Stenmark H. (2009) Rab GTPases as coordinators of vesicle traffic. Nat. Rev. Mol. Cell Biol. 10, 513–525 10.1038/nrm2728 19603039

